# (*E*)-4-Meth­oxy-2-(*p*-tolyl­imino­meth­yl)phenol

**DOI:** 10.1107/S1600536810003028

**Published:** 2010-01-30

**Authors:** Başak Koşar, Arzu Özek, Çiğdem Albayrak, Orhan Büyükgüngör

**Affiliations:** a Faculty of Education, Sinop University, Sinop, Turkey; b Department of Physics, Ondokuz Mayıs University, TR-55139 Samsun, Turkey

## Abstract

The mol­ecule of the title compound, C_15_H_15_NO_2_, adopts the enol–imine tautomeric form and has a strong intra­molecular O—H⋯N hydrogen bond as a result. The mol­ecule is almost planar, with a maximum deviation of 0.1038 (15) Å for the meth­oxy C atom. A weak C—H⋯π inter­action and a weak C—H⋯O hydrogen bond are present in the crystal.

## Related literature

For background to thermochromic Schiff bases, see: Moustakali-Mavridis *et al.* (1980 ▶). For related structures, see: Koşar *et al.* (2009 ▶); Tanak & Yavuz (2010 ▶).
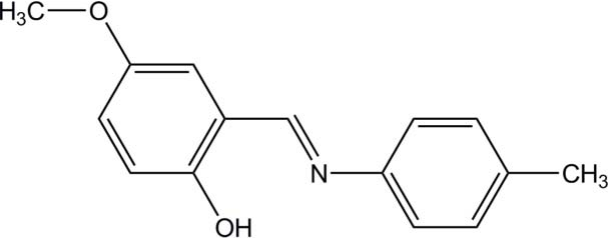


## Experimental

### 

#### Crystal data

C_15_H_15_NO_2_
*M* *_r_* = 241.28
Monoclinic, 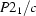

*a* = 21.1680 (9) Å
*b* = 4.7844 (2) Å
*c* = 12.2759 (4) Å
β = 92.859 (3)°
*V* = 1241.71 (8) Å^3^
*Z* = 4
Mo *K*α radiation
μ = 0.09 mm^−1^
*T* = 296 K
0.76 × 0.52 × 0.19 mm

#### Data collection

Stoe IPDS 2 diffractometer
Absorption correction: integration (*X-RED32*; Stoe & Cie, 2002 ▶) *T*_min_ = 0.947, *T*_max_ = 0.984
16465 measured reflections
2627 independent reflections
2223 reflections with *I* > 2σ(*I*)
*R*_int_ = 0.029

#### Refinement

*R*[*F* ^2^ > 2σ(*F* ^2^)] = 0.043
*wR*(*F* ^2^) = 0.127
*S* = 1.08
2627 reflections
169 parameters
H atoms treated by a mixture of independent and constrained refinement
Δρ_max_ = 0.18 e Å^−3^
Δρ_min_ = −0.17 e Å^−3^

### 

Data collection: *X-AREA* (Stoe & Cie, 2002 ▶); cell refinement: *X-AREA*; data reduction: *X-RED32* (Stoe & Cie, 2002 ▶); program(s) used to solve structure: *SHELXS97* (Sheldrick, 2008 ▶); program(s) used to refine structure: *SHELXL97* (Sheldrick, 2008 ▶); molecular graphics: *ORTEP-3 for Windows* (Farrugia, 1997 ▶); software used to prepare material for publication: *WinGX* (Farrugia, 1999 ▶).

## Supplementary Material

Crystal structure: contains datablocks I, global. DOI: 10.1107/S1600536810003028/is2517sup1.cif

Structure factors: contains datablocks I. DOI: 10.1107/S1600536810003028/is2517Isup2.hkl

Additional supplementary materials:  crystallographic information; 3D view; checkCIF report

## Figures and Tables

**Table 1:** Hydrogen-bond geometry (Å, °) *Cg*1 is the centroid of the C9–C14 ring.

*D*—H⋯*A*	*D*—H	H⋯*A*	*D*⋯*A*	*D*—H⋯*A*
O1—H1⋯N1	0.93 (2)	1.76 (2)	2.6178 (14)	151 (2)
C15—H15*C*⋯*Cg*1^i^	0.96	2.66	3.5535 (16)	156
C7—H7*B*⋯O2^ii^	0.96	2.57	3.496 (2)	163

Symmetry codes: (i) 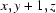; (ii) 
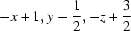
.

**Fig. 1.**
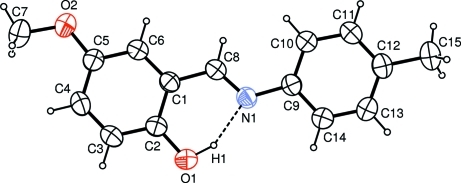
A view of the title compound, with the atom-numbering scheme. Displacement ellipsoids are drawn at 50% probability and dashed line indicates intramolecular hydrogen bond.

**Fig. 2.**
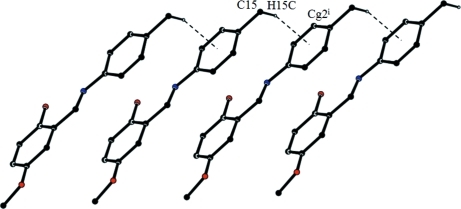
A partial packing diagram for crystal structure, showing the C—H···π bonds as dashed lines. [Symmetry code: (i) x, y + 1, z.]

**Fig. 3.**
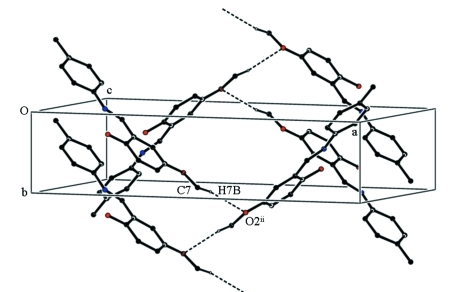
A partial packing diagram for crystal structure, showing the C—H···O intermolecular bonds as dashed lines. [Symmetry code: (ii) -x + 1, y - 1/2, -z + 3/2.]

## supplementary crystallographic information

### Comment

Schiff bases are formed by reaction of a primary amine and an aldehyde and have a wide area of usage as ligands in coordination chemistry. Especially *o*-hydroxy Schiff base derivatives are important classes and have attracted the interest of chemists and physicist because of their photochromic and thermochromic features in the solid state. These features are caused by the proton transfer to N atom from O atom with light in photochromic or with temperature in thermochromic Schiff bases. It has been claimed that the molecules showing thermochromism are planar and showing photochromism are non-planar (Moustakali-Mavridis *et al.*, 1980). In general, *o*-hydroxy Schiff bases can be found at two possible tautomeric forms called as phenol-imine and keto-amine. Depending on these tautomers, two different types of intramolecular hydrogen bonding are visible in *o*-hydroxy Schiff bases: O—H···N in phenol-imine and N—H···O in keto-amine tautomers.

The molecular structure of the title compound is almost planar with maximum deviation of 0.1038 (15) Å for methyl C7 and exhibits an enol-imine tautomeric form, as indicated by the following bond lengths: C8═N1 [1.2757 (15) Å], C1—C8 [1.4514 (16) Å] and C2—O1 [1.3509 (15) Å]. These bond lengths are in a good agreement with observed for (*E*)-2-[(4-chlorophenyl)iminomethyl]-5-methoxyphenol [1.282 (2), 1.436 (2) and 1.3452 (18) Å; Koşar *et al.*, 2009], which is also an enol-imine tautomer. The same bond lengths are comparable with observed for (*E*)-2-[(2-hydroxy-5-nitrophenyl)-iminomethyl]-4-nitrophenolate (1.288, 1.420 and 1.2749 Å; Tanak & Yavuz, 2010), which is a keto-amine tautomer.

As a result of enol-imine form of the molecule, there is a strong intramolecular hydrogen bond between O1 and N1 (Fig. 1). The three dimensional crystal structure is stabilized by one weak C—H···π interaction (*Cg*1 is the centroid of the C9–C14 ring) and one weak C—H···O hydrogen bond (between C7 and O2 of neighbor molecule) and van der Waals interactions (Figs. 2 & 3). Both intramolecular and intermolecular hydrogen bonding geometries can be seen in Table 1.

### Experimental

The compound (*E*)-4-methoxy-2-[(*p*-tolylimino)methyl]phenol was prepared by reflux a mixture of a solution containing 5-methoxysalicylaldehyde (0.5 g, 3.3 mmol) in 20 ml ethanol and a solution containing 4-methylaniline (0.35 g, 3.3 mmol) in 20 ml ethanol. The reaction mixture was stirred for 1 h under reflux. The crystals of (*E*)-4-methoxy-2-[(*p*-tolylimino)methyl]phenol suitable for X-ray analysis were obtained from benzene by slow evaporation (yield 81%; m.p. 377–378 K).

### Refinement

All H atoms except for H1 were refined using a riding model, with C—H distances of 0.96 Å for the methyl group and 0.93 Å for the aromatic groups. The displacement parameters of these H atoms were fixed at 1.2*U*_eq_ of their parent carbon atom for the aromatic groups and 1.5*U*_eq_ of their parent atoms for the methyl group.

### Crystal data

**Table:**

C_15_H_15_NO_2_	*F*(000) = 512
*M**_r_* = 241.28	*D*_x_ = 1.291 Mg m^−^^3^
Monoclinic, *P*2_1_/*c*	Mo *K*α radiation, λ = 0.71073 Å
Hall symbol: -P 2ybc	Cell parameters from 16465 reflections
*a* = 21.1680 (9) Å	θ = 1.7–27.3°
*b* = 4.7844 (2) Å	µ = 0.09 mm^−^^1^
*c* = 12.2759 (4) Å	*T* = 296 K
β = 92.859 (3)°	Prism, brown
*V* = 1241.71 (8) Å^3^	0.76 × 0.52 × 0.19 mm
*Z* = 4	

### Data collection

**Table:**

Stoe IPDS 2 diffractometer	2627 independent reflections
Radiation source: fine-focus sealed tube	2223 reflections with *I* > 2σ(*I*)
graphite	*R*_int_ = 0.029
Detector resolution: 6.67 pixels mm^-1^	θ_max_ = 26.8°, θ_min_ = 1.9°
ω scans	*h* = −26→26
Absorption correction: integration (*X-RED32*; Stoe & Cie, 2002)	*k* = −6→6
*T*_min_ = 0.947, *T*_max_ = 0.984	*l* = −15→15
16465 measured reflections	

### Refinement

**Table:**

Refinement on *F*^2^	Primary atom site location: structure-invariant direct methods
Least-squares matrix: full	Secondary atom site location: difference Fourier map
*R*[*F*^2^ > 2σ(*F*^2^)] = 0.043	Hydrogen site location: inferred from neighbouring sites
*wR*(*F*^2^) = 0.127	H atoms treated by a mixture of independent and constrained refinement
*S* = 1.08	*w* = 1/[σ^2^(*F*_o_^2^) + (0.0679*P*)^2^ + 0.1706*P*] where *P* = (*F*_o_^2^ + 2*F*_c_^2^)/3
2627 reflections	(Δ/σ)_max_ < 0.001
169 parameters	Δρ_max_ = 0.18 e Å^−^^3^
0 restraints	Δρ_min_ = −0.17 e Å^−^^3^

### Special details

**Table:**

Experimental. 331 frames, detector distance = 120 mm
Geometry. All e.s.d.'s (except the e.s.d. in the dihedral angle between two l.s. planes) are estimated using the full covariance matrix. The cell e.s.d.'s are taken into account individually in the estimation of e.s.d.'s in distances, angles and torsion angles; correlations between e.s.d.'s in cell parameters are only used when they are defined by crystal symmetry. An approximate (isotropic) treatment of cell e.s.d.'s is used for estimating e.s.d.'s involving l.s. planes.
Refinement. Refinement of *F*^2^ against ALL reflections. The weighted *R*-factor *wR* and goodness of fit *S* are based on *F*^2^, conventional *R*-factors *R* are based on *F*, with *F* set to zero for negative *F*^2^. The threshold expression of *F*^2^ > σ(*F*^2^) is used only for calculating *R*-factors(gt) *etc*. and is not relevant to the choice of reflections for refinement. *R*-factors based on *F*^2^ are statistically about twice as large as those based on *F*, and *R*- factors based on ALL data will be even larger.

### Fractional atomic coordinates and isotropic or equivalent isotropic displacement parameters (Å^2^)

**Table:**

	*x*	*y*	*z*	*U*_iso_*/*U*_eq_	
C1	0.30136 (5)	0.2222 (2)	0.54620 (9)	0.0391 (3)	
C2	0.30433 (6)	0.1581 (3)	0.43466 (9)	0.0450 (3)	
C3	0.34823 (7)	−0.0358 (3)	0.40249 (10)	0.0548 (4)	
H3	0.3503	−0.0789	0.3289	0.066*	
C4	0.38887 (6)	−0.1658 (3)	0.47753 (11)	0.0512 (3)	
H4	0.4183	−0.2941	0.4543	0.061*	
C5	0.38594 (6)	−0.1055 (3)	0.58806 (10)	0.0454 (3)	
C6	0.34203 (6)	0.0854 (3)	0.62107 (10)	0.0442 (3)	
H6	0.3395	0.1235	0.6950	0.053*	
C7	0.47395 (8)	−0.3961 (4)	0.63806 (15)	0.0720 (5)	
H7A	0.4571	−0.5564	0.5996	0.108*	
H7B	0.4985	−0.4560	0.7017	0.108*	
H7C	0.5004	−0.2923	0.5913	0.108*	
C8	0.25662 (5)	0.4249 (3)	0.58486 (9)	0.0420 (3)	
H8	0.2549	0.4552	0.6595	0.050*	
C9	0.17663 (5)	0.7627 (2)	0.55871 (9)	0.0400 (3)	
C10	0.17533 (6)	0.8537 (3)	0.66634 (10)	0.0491 (3)	
H10	0.2043	0.7827	0.7187	0.059*	
C11	0.13125 (6)	1.0488 (3)	0.69553 (11)	0.0503 (3)	
H11	0.1312	1.1069	0.7678	0.060*	
C12	0.08726 (6)	1.1606 (3)	0.62089 (11)	0.0450 (3)	
C13	0.08968 (6)	1.0724 (3)	0.51344 (11)	0.0518 (3)	
H13	0.0610	1.1455	0.4610	0.062*	
C14	0.13368 (6)	0.8786 (3)	0.48282 (10)	0.0488 (3)	
H14	0.1345	0.8250	0.4101	0.059*	
C15	0.03942 (7)	1.3726 (3)	0.65439 (13)	0.0572 (4)	
H15A	0.0372	1.3706	0.7323	0.086*	
H15B	−0.0013	1.3271	0.6211	0.086*	
H15C	0.0519	1.5551	0.6311	0.086*	
N1	0.21955 (5)	0.5626 (2)	0.51970 (8)	0.0419 (3)	
O1	0.26517 (5)	0.2806 (3)	0.35863 (7)	0.0651 (3)	
O2	0.42372 (5)	−0.2245 (2)	0.66971 (8)	0.0639 (3)	
H1	0.2419 (10)	0.410 (5)	0.3970 (18)	0.103 (7)*	

### Atomic displacement parameters (Å^2^)

**Table:**

	*U*^11^	*U*^22^	*U*^33^	*U*^12^	*U*^13^	*U*^23^
C1	0.0390 (6)	0.0401 (6)	0.0385 (6)	−0.0012 (5)	0.0060 (4)	−0.0015 (5)
C2	0.0475 (6)	0.0508 (7)	0.0370 (6)	0.0051 (5)	0.0050 (5)	0.0012 (5)
C3	0.0614 (8)	0.0640 (9)	0.0398 (6)	0.0124 (7)	0.0105 (6)	−0.0048 (6)
C4	0.0478 (7)	0.0540 (8)	0.0527 (7)	0.0110 (6)	0.0108 (6)	−0.0052 (6)
C5	0.0430 (6)	0.0469 (7)	0.0463 (6)	0.0032 (5)	0.0009 (5)	0.0010 (5)
C6	0.0461 (6)	0.0484 (7)	0.0382 (6)	0.0032 (5)	0.0028 (5)	−0.0037 (5)
C7	0.0635 (9)	0.0716 (10)	0.0799 (10)	0.0261 (8)	−0.0057 (8)	−0.0009 (8)
C8	0.0441 (6)	0.0438 (6)	0.0384 (6)	0.0015 (5)	0.0045 (5)	−0.0033 (5)
C9	0.0389 (6)	0.0384 (6)	0.0429 (6)	−0.0010 (5)	0.0049 (4)	−0.0010 (5)
C10	0.0505 (7)	0.0535 (7)	0.0430 (6)	0.0096 (6)	−0.0008 (5)	−0.0044 (5)
C11	0.0550 (7)	0.0505 (7)	0.0458 (6)	0.0047 (6)	0.0063 (5)	−0.0074 (6)
C12	0.0427 (6)	0.0358 (6)	0.0573 (7)	−0.0022 (5)	0.0104 (5)	0.0002 (5)
C13	0.0520 (7)	0.0495 (7)	0.0535 (7)	0.0096 (6)	−0.0026 (6)	0.0037 (6)
C14	0.0541 (7)	0.0499 (7)	0.0422 (6)	0.0067 (6)	0.0017 (5)	−0.0014 (5)
C15	0.0541 (7)	0.0439 (7)	0.0748 (9)	0.0063 (6)	0.0152 (7)	−0.0006 (6)
N1	0.0421 (5)	0.0425 (5)	0.0416 (5)	0.0025 (4)	0.0053 (4)	−0.0013 (4)
O1	0.0743 (7)	0.0839 (8)	0.0369 (5)	0.0305 (6)	−0.0001 (4)	−0.0002 (5)
O2	0.0612 (6)	0.0753 (7)	0.0545 (6)	0.0256 (5)	−0.0038 (5)	0.0009 (5)

### Geometric parameters (Å, °)

**Table:**

C1—C6	1.3914 (17)	C8—H8	0.9300
C1—C2	1.4077 (16)	C9—C14	1.3846 (17)
C1—C8	1.4514 (16)	C9—C10	1.3928 (17)
C2—O1	1.3509 (15)	C9—N1	1.4194 (15)
C2—C3	1.3843 (18)	C10—C11	1.3798 (18)
C3—C4	1.3772 (19)	C10—H10	0.9300
C3—H3	0.9300	C11—C12	1.3811 (18)
C4—C5	1.3917 (18)	C11—H11	0.9300
C4—H4	0.9300	C12—C13	1.3882 (19)
C5—O2	1.3741 (15)	C12—C15	1.5050 (17)
C5—C6	1.3782 (17)	C13—C14	1.3796 (18)
C6—H6	0.9300	C13—H13	0.9300
C7—O2	1.4130 (17)	C14—H14	0.9300
C7—H7A	0.9600	C15—H15A	0.9600
C7—H7B	0.9600	C15—H15B	0.9600
C7—H7C	0.9600	C15—H15C	0.9600
C8—N1	1.2757 (15)	O1—H1	0.93 (2)
			
C6—C1—C2	118.93 (11)	C14—C9—C10	118.01 (11)
C6—C1—C8	119.39 (10)	C14—C9—N1	116.94 (10)
C2—C1—C8	121.68 (11)	C10—C9—N1	125.04 (11)
O1—C2—C3	119.41 (11)	C11—C10—C9	120.23 (12)
O1—C2—C1	121.45 (11)	C11—C10—H10	119.9
C3—C2—C1	119.13 (11)	C9—C10—H10	119.9
C4—C3—C2	121.17 (12)	C10—C11—C12	122.14 (12)
C4—C3—H3	119.4	C10—C11—H11	118.9
C2—C3—H3	119.4	C12—C11—H11	118.9
C3—C4—C5	120.12 (12)	C11—C12—C13	117.20 (11)
C3—C4—H4	119.9	C11—C12—C15	121.35 (12)
C5—C4—H4	119.9	C13—C12—C15	121.44 (12)
O2—C5—C6	115.88 (11)	C14—C13—C12	121.37 (12)
O2—C5—C4	124.94 (11)	C14—C13—H13	119.3
C6—C5—C4	119.18 (12)	C12—C13—H13	119.3
C5—C6—C1	121.45 (11)	C13—C14—C9	121.02 (12)
C5—C6—H6	119.3	C13—C14—H14	119.5
C1—C6—H6	119.3	C9—C14—H14	119.5
O2—C7—H7A	109.5	C12—C15—H15A	109.5
O2—C7—H7B	109.5	C12—C15—H15B	109.5
H7A—C7—H7B	109.5	H15A—C15—H15B	109.5
O2—C7—H7C	109.5	C12—C15—H15C	109.5
H7A—C7—H7C	109.5	H15A—C15—H15C	109.5
H7B—C7—H7C	109.5	H15B—C15—H15C	109.5
N1—C8—C1	122.08 (11)	C8—N1—C9	121.40 (10)
N1—C8—H8	119.0	C2—O1—H1	105.0 (13)
C1—C8—H8	119.0	C5—O2—C7	117.30 (11)
			
C6—C1—C2—O1	178.73 (12)	C14—C9—C10—C11	1.5 (2)
C8—C1—C2—O1	−0.64 (19)	N1—C9—C10—C11	−179.72 (11)
C6—C1—C2—C3	−1.02 (19)	C9—C10—C11—C12	0.0 (2)
C8—C1—C2—C3	179.61 (12)	C10—C11—C12—C13	−1.2 (2)
O1—C2—C3—C4	−179.83 (13)	C10—C11—C12—C15	179.81 (12)
C1—C2—C3—C4	−0.1 (2)	C11—C12—C13—C14	0.8 (2)
C2—C3—C4—C5	0.6 (2)	C15—C12—C13—C14	179.82 (12)
C3—C4—C5—O2	179.39 (13)	C12—C13—C14—C9	0.7 (2)
C3—C4—C5—C6	0.0 (2)	C10—C9—C14—C13	−1.9 (2)
O2—C5—C6—C1	179.43 (11)	N1—C9—C14—C13	179.26 (12)
C4—C5—C6—C1	−1.1 (2)	C1—C8—N1—C9	−179.19 (10)
C2—C1—C6—C5	1.62 (19)	C14—C9—N1—C8	−173.10 (11)
C8—C1—C6—C5	−179.00 (11)	C10—C9—N1—C8	8.12 (19)
C6—C1—C8—N1	177.85 (11)	C6—C5—O2—C7	−172.88 (13)
C2—C1—C8—N1	−2.79 (19)	C4—C5—O2—C7	7.7 (2)

### Hydrogen-bond geometry (Å, °)

**Table:**

Cg1 is the centroid of the C9–C14 ring.

**Table:**

*D*—H···*A*	*D*—H	H···*A*	*D*···*A*	*D*—H···*A*
O1—H1···N1	0.93 (2)	1.76 (2)	2.6178 (14)	151 (2)
C15—H15C···Cg1^i^	0.96	2.66	3.5535 (16)	156
C7—H7B···O2^ii^	0.96	2.57	3.496 (2)	163

Symmetry codes: (i) *x*, *y*+1, *z*; (ii) −*x*+1, *y*−1/2, −*z*+3/2.
